# Cartilage in facet joints of patients with ankylosing spondylitis (AS) shows signs of cartilage degeneration rather than chondrocyte hypertrophy: implications for joint remodeling in AS

**DOI:** 10.1186/s13075-015-0675-5

**Published:** 2015-07-17

**Authors:** Janine Bleil, Joachim Sieper, Rene Maier, Uwe Schlichting, Axel Hempfing, Uta Syrbe, Heiner Appel

**Affiliations:** Charité Universitätsmedizin Berlin, Campus Benjamin Franklin, Medizinische Klinik für Gastroenterologie, Infektiologie und Rheumatologie, Hindenburgdamm 30, 12203 Berlin, Germany; Werner-Wicker-Klinik, Im Kreuzfeld 4, 34537 Bad Wildungen, Germany; Charité Universitätsmedizin Berlin, Institut für Pathologie, Charitéplatz 1, 10117 Berlin, Germany; Deutsches Rheuma-Forschungszentrum, Charitéplatz 1, 10117 Berlin, Germany

## Abstract

**Introduction:**

In ankylosing spondylitis (AS), joint remodeling leading to joint ankylosis involves cartilage fusion. Here, we analyzed whether chondrocyte hypertrophy is involved in cartilage fusion and subsequent joint remodeling in AS.

**Methods:**

We assessed the expression of chondrocyte hypertrophy markers runt-related transcription factor 2 (Runx2), type X collagen (COL10), matrix metalloproteinase 13 (MMP13), osteocalcin and beta-catenin and the expression of positive bone morphogenic proteins (BMPs) and negative regulators (dickkopf-1 (DKK-1)), sclerostin, (wingless inhibitory factor 1 (wif-1)) of chondrocyte hypertrophy in the cartilage of facet joints from patients with AS or osteoarthritis (OA) and from autopsy controls (CO) by immunohistochemistry. Sex determining region Y (SRY)-box 9 (Sox9) and type II collagen (COL2) expression was assessed as indicators of chondrocyte integrity and function.

**Results:**

The percentage of hypertrophic chondrocytes expressing Runx2, COL10, MMP13, osteocalcin or beta-catenin was significantly increased in OA but not in AS joints compared to CO joints. Frequencies of sclerostin-positive and DKK-1-positive chondrocytes were similar in AS and CO. In contrast, wif-1- but also BMP-2- and BMP-7-expressing and Sox9-expressing chondrocytes were drastically reduced in AS joints compared to CO as well as OA joints whereas the percentage of COL2-expressing chondrocytes was significantly higher in AS joints compared to CO joints.

**Conclusions:**

We found no evidence for chondrocyte hypertrophy within hyaline cartilage of AS joints even in the presence of reduced expression of the *wnt* inhibitor wif-1 suggesting that chondrocyte hypertrophy is not a predominant pathway involved in joint fusion and remodeling in AS. In contrast, the reduced expression of Sox9, BMP-2 and BMP-7 concomitantly with induced COL2 expression rather point to disturbed cartilage homeostasis promoting cartilage degeneration in AS.

## Introduction

Ankylosing spondylitis (AS) is a chronic inflammatory disease primarily affecting the sacroiliac joints and the spine [1]. Typically, inflammation is followed by new bone formation, which can lead to joint ankylosis, for instance of the sacroiliac joints and the spine.

Although being a prominent sign of the disease, the mechanisms promoting joint ankylosis in AS are poorly defined. Capsular ankylosis and development of extraarticular osteophytes has been reported in AS joints in several studies [2, 3]. However, our histomorphometric study as well as a study in which we performed computed tomography (CT) scans of the facet, i.e., zygapophyseal joints provided no evidence for a major contribution of capsular ankylosis or extraarticular osteophytes in AS facet joints [4]. In fact we [4] and others [5] observed that joint ankylosis is initiated by fusion of both cartilaginous surfaces, which is followed by bony intraarticular ankylosis. In our study, AS facet joints were grouped into joints according to progressive loss of joint space and the type of joint fusion into joints with open joint space (stage I), joints with cartilaginous fusion (stage II) and joints with bony fusion (stage III) [4]. Further histomorphometric analysis indicated that progressive remodeling is accompanied by thinning of the cartilage and by cartilage degeneration involving chondrocyte apoptosis and proteoglycan loss [4]. Moreover, the coincidental occurrence of a subchondral fibrous tissue, which carried bone-destructive features, with cartilaginous joint fusion suggested a major contribution of this tissue to the remodeling process.

In the transgenic tumor necrosis factor (TNF) mouse model, which is characterized by inflammation in multiple joints including the sacroiliac joints, intraarticular joint fusion and ankylosis in the sacroiliac joints can be induced by blockade of dickkopf-1 (DKK-1) – an antagonist of the wingless (*wnt*) pathway [6]. Joint fusion and ankylosis in this setting is mediated via the induction of chondrocyte hypertrophy that leads to reinitiation of the endochondral bone formation pathway, which eventually leads to bone synthesis and simultaneous loss of cartilage.

Chondrocyte hypertrophy is induced by activation of the *wnt* pathway leading to intracellular beta-catenin accumulation in chondrocytes [7, 8]. This promotes upregulation of the runt-related transcription factor 2 (Runx2) [9–12] and induction of matrix metalloproteinase 13 (MMP13) [13, 14] and type X collagen (COL10) [15, 16]. In particular, MMP13 and COL10 are considered typical markers of hypertrophic chondrocytes. Differentiation of chondrocytes is controlled by endogenous regulators of the *wnt* pathway and by growth factors. DKK-1 and sclerostin but also wingless inhibitory factor 1 (wif-1) are negative regulators of the *wnt* pathway while bone morphogenic proteins (BMPs) are growth factors, which are important for cartilage homeostasis on one hand [17, 18] while their overexpression can promote chondrogenesis and chondrocyte hypertrophy [19]. Endochondral bone formation and chondrocyte hypertrophy are involved in bone development and longitudinal growth of long bones [20]. With the disappearance of growth plates at the end of the second decade of life, chondrocyte hypertrophy is usually not seen in joints of adults [21, 22]. However, under pathological conditions such as in osteoarthritis (OA), a disease that is also associated with osteoproliferation, chondrocytes can reacquire a hypertrophic phenotype, which may promote osteophyte development and cartilage degeneration [21, 23–25].

To determine if chondrocyte hypertrophy is involved in joint fusion and remodeling in AS joints, we analyzed the expression of markers of chondrocyte hypertrophy and of regulators of chondrocyte hypertrophy in facet joints of AS patients. The results were compared to joints of controls without joint disease and OA patients.

## Methods

### Patients and tissue acquisition

A total of 17 facet joints from 14 patients with AS (12 male, 2 female; mean age ± standard deviation (SD) 51 ± 8.12 years) undergoing surgical correction of rigid hyperkyphosis were included in the analysis. These joints belonged to stages I–III of AS joint remodeling as previously described by us. Joints without cartilage, due to complete replacement of the joint by trabecular bone, were excluded from this study. Of 14 AS patients, 11 were treated with nonsteroidal anti-inflammatory drugs (NSAIDs), none of the AS patients received disease-modifying antirheumatic drugs or TNF-alpha blocking agents at the time of tissue acquisition. In addition, 22 facet joints from 12 patients with OA (1 male, 11 female; mean age ± SD 69.83 ± 6.01 years) who underwent surgery of the lumbar spine because of neurological deficits in the lower limbs caused by compression of nerve roots were acquired and 11 facet joints of 10 non-AS control patients (4 male, 6 female; mean age ± SD 68.90 ± 14.91 years) who had no history of rheumatic diseases and died of cardiovascular diseases, were removed in toto at autopsy.

All patients gave informed consent to the study. Permission for this study was given by the local ethics committee of the Charité University Medicine Berlin, Campus Benjamin Franklin, Berlin, Germany.

### Tissue preparation

After acquisition, all joints were fixed, decalcified with ethylenediaminetetraacetic acid and embedded in paraffin as described [4]. Sections 4–6 μm thick were used for stainings. For overview the sections were stained using safranin O/light green.

### Immunohistochemistry

For immunohistochemistry, fixed, decalcified tissue sections were deparaffinized in xylene and rehydrated before either heat-induced epitope retrieval with citrate buffer at pH 6.0 for beta-catenin, BMP-2, BMP-7 and wif-1 detection or enzymatic retrieval for sclerostin, DKK-1, Runx2, osteocalcin, sex determining region Y (SRY)-box 9 (Sox9), type II collagen (COL2) and COL10 detection. After blocking of nonspecific binding by serum-free protein block (Dako, Glostrup, Denmark) sections were incubated with the respective primary antibodies overnight at 4 °C. After blocking with an endogenous avidin/biotin-blocking kit (Invitrogen, Paisley, UK), the slides were incubated with species-specific biotinylated immunoglobulin (Dianova, Hamburg, Germany) and alkaline phosphatase streptavidin (Vector Laboratories, Burlingame, CA, USA). Alkaline phosphatase was visualized using Chromogen Red (Dako REAL Detection System Kit, Dako, Glostrup, Denmark) before counterstaining with Meyer’s hematoxylin.

#### Primary antibodies

Monoclonal mouse antibodies against osteocalcin (clone 190125, dilution 1:100; R&D Systems, Minneapolis, MN, USA), wif-1 (clone 133015, dilution 1:30; R&D Systems, Minneapolis, MN, USA), COL2 (clone II-4C11, dilution 1:75; Acris Antibodies GmbH, Herford, Germany), COL10 (clone X53, dilution 1:25; Quartett, Berlin, Germany), polyclonal rabbit antibodies against beta-catenin (dilution 1:50; Thermo Fisher Scientific, Waltham, MA, USA), DKK-1 (dilution 1:50; Abcam, Cambridge, UK), sclerostin (dilution 1:100; Abcam, Cambridge, UK), Runx2 (dilution 1:40; Quartett, Berlin, Germany), BMP-2 and BMP-7 (dilution 1:20; PeproTech, Rocky Hill, NJ, USA) and polyclonal goat antibodies against MMP13 (dilution 1:20) and Sox9 (dilution 1:20) both obtained from R&D Systems (Minneapolis, MN, USA) were used.

As negative controls, experiments were performed (i) with isotype controls for immunoglobulin G (IgG) (mouse IgG1 DAK-GO1, mouse IgG2a DAK-GO5, mouse IgG2b DAK-GO9 and rabbit IgG all Dako, Glostrup, Denmark) and (ii) by omitting the primary antibodies.

The expression of the respective markers was evaluated by calculating the percentage of positively stained chondrocytes, i.e., the number of positively stained chondrocytes per total number of chondrocytes. The joint sections of AS patients contained 2635 ± 397 chondrocytes (mean ± SD) and as a minimum 337 chondrocytes, joint sections of OA patients 1461 ± 296 chondrocytes (minimum 332 chondrocytes) and sections of autopsy controls (CO) 2294 ± 517 (minimum 589) chondrocytes. The cartilage area was defined beforehand by safranin O staining.

#### Microscope, camera and software

Immunohistological analysis was performed using an Olympus BX60 microscope (Hamburg, Germany). Pictures were taken with a digital camera (Color View II; Soft Imaging System, Hamburg, Germany) and analyzed using image analysis software (Soft Imaging Software Cell D, Olympus Soft Imaging Solutions GmbH, Hamburg, Germany).

#### Statistical analysis

Statistical analyses were performed using GraphPad Prism software (GraphPad Prism 5 for Windows, Version 5.01, GraphPad Software Inc., San Diego, CA, USA). Individual measurements and the median are shown in the graphs. For multiple group comparisons the Kruskal-Wallis test and the Dunn’s multiple comparison test as post test were used; a *p* value less than 0.05 was considered significant. For correlation analysis the Spearman correlation coefficient was calculated.

## Results

### No increased percentage of hypertrophic chondrocytes expressing Runx2, COL10 or MMP13 within the hyaline articular cartilage in AS facet joints

**Fig. 1:**
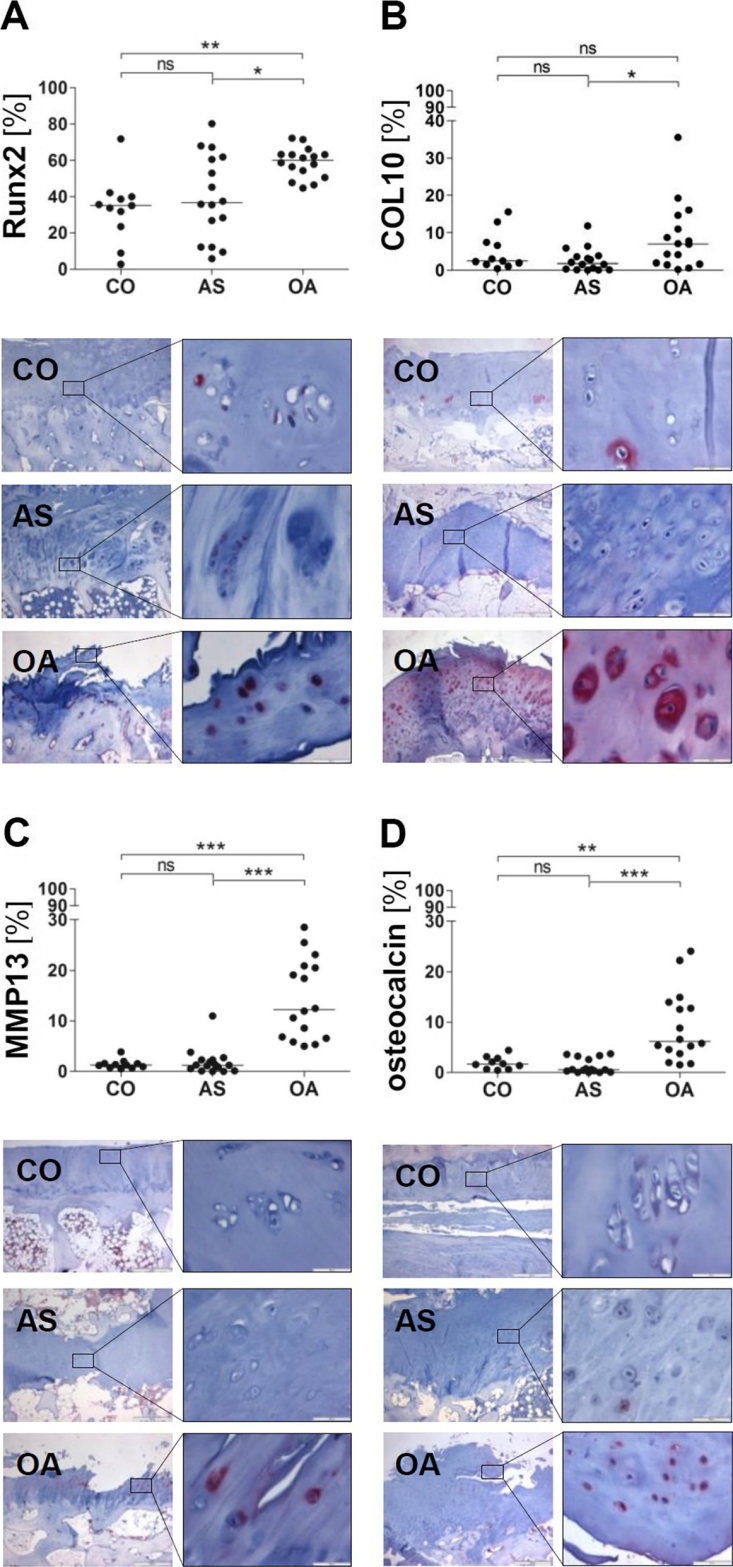
No increased percentage of hypertrophic chondrocytes expressing Runx2, COL10 or MMP13 within the hyaline articular cartilage in ankylosing spondylitis facet joints. The percentage of positively stained chondrocytes among all chondrocytes [%] and representative immunohistochemical staining of Runx2 (**a**), COL10 (**b**), MMP13 (**c**) and osteocalcin (**d**) within the hyaline articular cartilage of facet joints from controls (CO; *A–D upper pictures*), patients with ankylosing spondylitis (AS; *A–D middle pictures*) and osteoarthritis (OA; *A–D lower pictures*); original magnification 40× and 400×; ns = not significant *p* >0.05; ***= *p* <0.001; **= *p* <0.01, *= *p* <0.05. *COL10* type X collagen, *MMP* matrix metalloproteinase, *Runx2*, runt-related transcription factor 2

While the number of chondrocytes expressing Runx2, COL10 and MMP13 was significantly increased in OA facet joints (mean ± SD: Runx2 = 58.8 ± 8.3 %, COL10 = 8.8 ± 9.2 %, MMP13 = 14.3 ± 7.9 %) compared to autopsy controls (CO) (Runx2 = 33.1 ± 18.1 %, COL10 = 4.9 ± 4.9 %, MMP13 = 1.4 ± 0.9 %), no increased frequencies of Runx2-, COL10- and MMP13-expressing chondrocytes were found in joints of AS patients (Runx2 = 40.0 ± 23.5 %, COL10 = 2.7 ± 3.2 %, MMP13 = 1.9 ± 2.6 %; Fig. 1a–c). In addition, the percentage of chondrocytes expressing osteocalcin was also significantly increased in OA joints (mean ± SD: 9.1 ± 7.0 %) but not in AS joints (mean ± SD: 1.3 ± 1.4 %) compared to CO (mean ± SD: 1.9 ± 1.3 %; Fig. 1d).

### No evidence for stage-dependent induction of chondrocyte hypertrophy

**Fig. 2:**
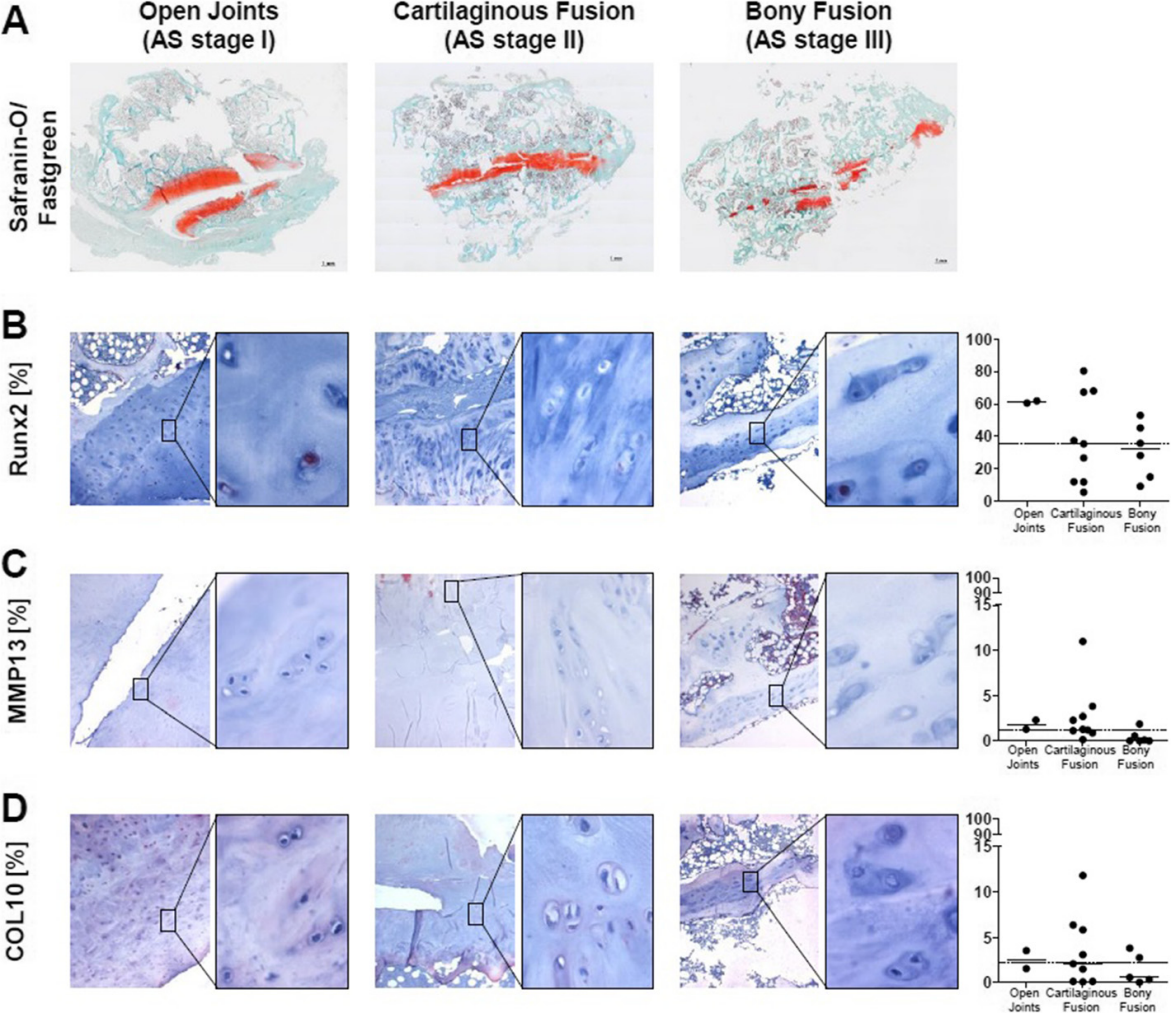
No evidence for stage-dependent induction of chondrocyte hypertrophy. Representative immunohistochemical staining of safranin-O/light green (**a**) from facet joints of patients with ankylosing spondylitis with open joints (AS stage I), with cartilaginous fused joints (AS stage II) and with bony fused joints (AS stage III). Representative immunohistochemical staining and percentage of chondrocytes that were positive for Runx2 (**b**), MMP13 (**c**) and COL10 (**d**) within the hyaline articular cartilage of facet joints from patients with AS stage I (*B–D first column*), AS stage II (*B–D second column*) and AS stage III (*B–D third column*); original magnification 40× and 400×; dashed line indicates the median of the control group. *COL10* type X collagen, *MMP* matrix metalloproteinase, *Runx2*, runt-related transcription factor 2

To determine if occurrence of chondrocyte hypertrophy is restricted to distinct stages of facet joint remodeling, which we have recently described [4], we also performed a subanalysis of AS joints grouped according to the proposed stages. Even though the low number of stage I joints precluded a statistical testing, Runx2 expression appeared higher in early stages of joint remodeling compared to controls. However, this was not accompanied by increased COL10 or MMP13 expression suggesting that chondrocyte hypertrophy also does not occur selectively in early stages of joint remodeling in AS (Fig. 2).

### No activation of the wnt pathway in chondrocytes in AS facet joints

**Fig. 3:**
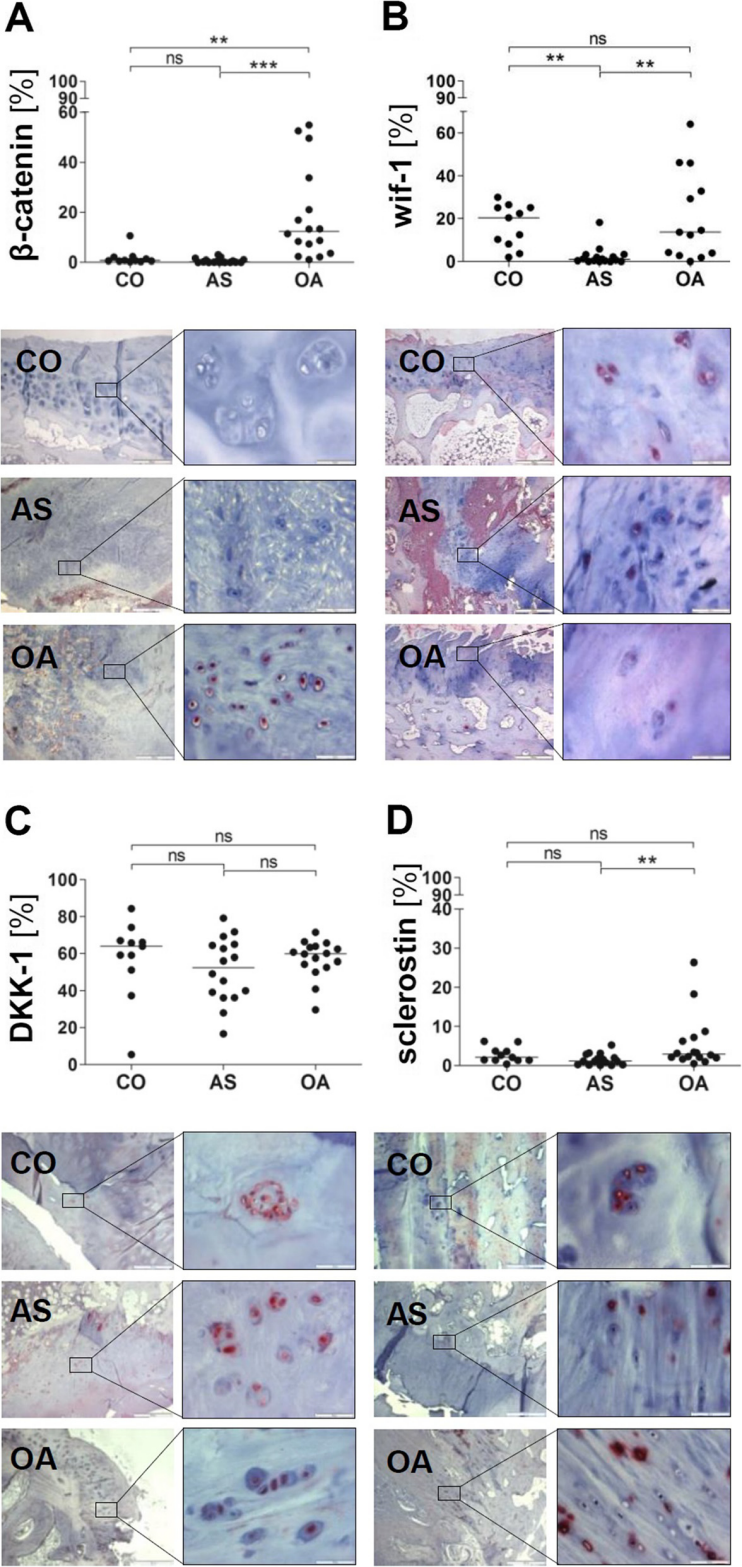
No activation of the *wnt* pathway in chondrocytes according to beta-catenin staining in ankylosing spondylitis facet joints. Percentage of positively stained chondrocytes [%] and representative immunohistochemical staining of beta-catenin (**a**), wif-1 (**b**), DKK-1 (**c**) and sclerostin (**d**) in the hyaline articular cartilage of facet joints from controls (CO; *A–D upper pictures*), patients with ankylosing spondylitis (AS; *A–D middle pictures*) and osteoarthritis (OA; *A–D lower pictures*); original magnification 40× and 400×; ns = not significant *p* >0.05; ***= *p* <0.001; **= *p* <0.01. *COL10* type X collagen, *DKK-1* dickkopf-1, *wif-1* wingless inhibitor factor-1, *wnt* wingless

We also analyzed the expression of beta-catenin, which is an indicator for the activation of the *wnt* pathway facilitating chondrocyte hypertrophy and the expression of inhibitors of the *wnt* pathway. In CO joints less than 2 % of chondrocytes expressed beta-catenin (mean ± SD: 1.8 ± 3.0 %). A low percentage of beta-catenin-positive chondrocytes was also found in AS facet joints (0.6 ± 0.9 %) while in OA joints the number of beta-catenin-positive chondrocytes was significantly increased (18.7 ± 18.5 %; Fig. 3a).

The percentage of chondrocytes expressing the *wnt* pathway inhibitor wif-1 was drastically reduced in AS joints (Fig. 3b; mean ± SD: wif-1 = 2.4 ± 4.5 %) compared to CO (wif-1 = 16.9 ± 9.9 %) and OA joints (wif-1 = 20.9 ± 20.8 %).

The percentage of DKK-1-positive chondrocytes was high, i.e., above 50 % and without difference between AS, OA and CO joints (mean ± SD: AS = 51.0 ± 17.5 %, OA = 57.2 ± 10.3 %, CO = 57.6 ± 21.1 %; Fig. 3c). The percentage of sclerostin-positive chondrocytes did not significantly differ between AS and CO joints (AS: mean ± SD: 1.5 ± 1.5 %; CO: mean ± SD: 2.7 ± 2.0 %; Fig. 3d) while the percentage of sclerostin-positive chondrocytes was increased in OA joints (mean ± SD: 5.7 ± 7.0 %). Correlation analysis showed that the number of sclerostin-positive chondrocytes correlated with the number of beta-catenin-positive chondrocytes in AS joints (r_s_ = 0.50; *p* <0.05) while in OA joints a correlation between the number of DKK-1-positive chondrocytes and the number of beta-catenin-positive chondrocytes was found (r_s_ = 0.45; *p* <0.05).

### Reduced expression of chondrocyte growth factors within the cartilage of AS joints

**Fig. 4:**
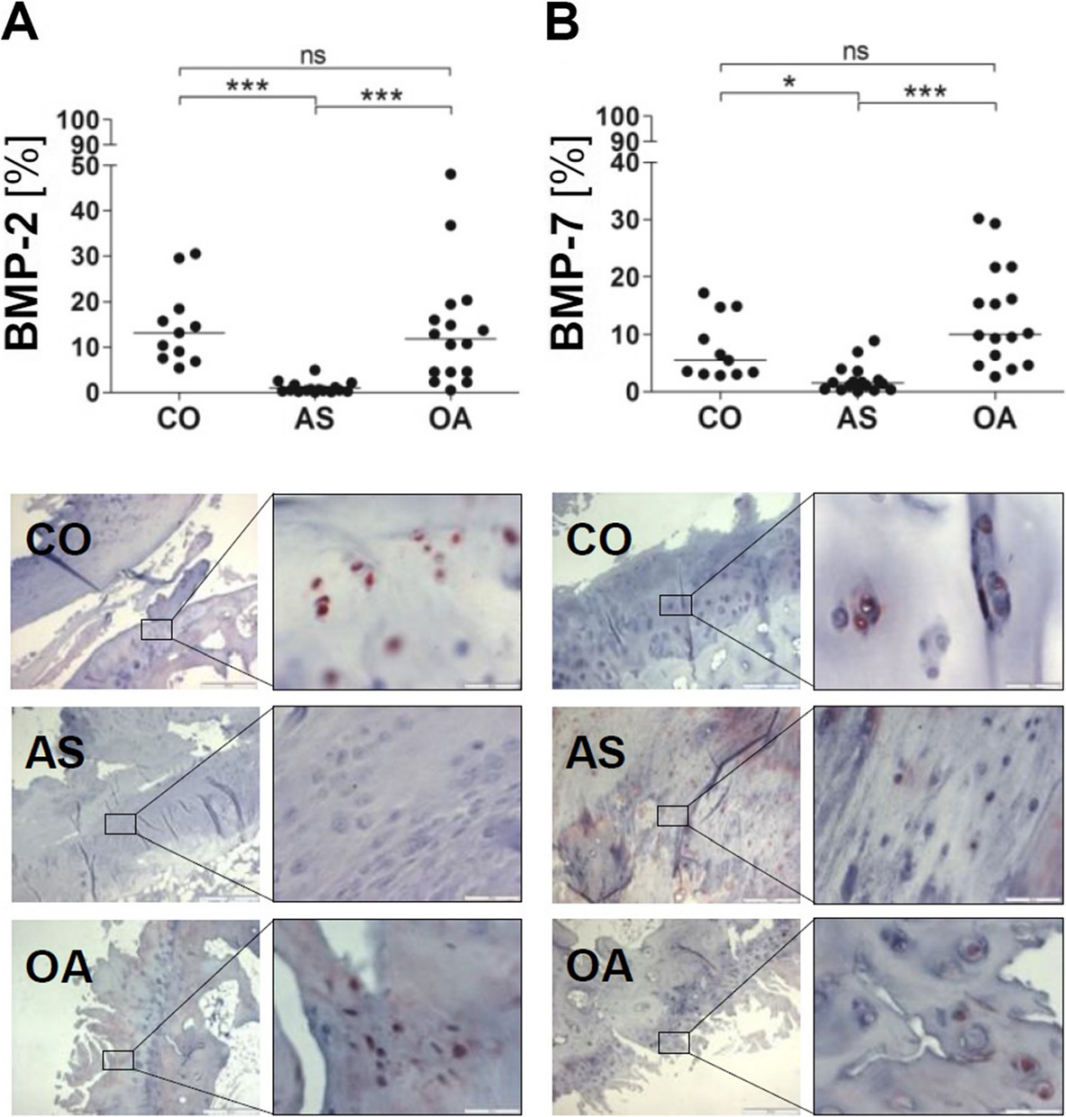
Reduced expression of chondrocyte growth factors within the cartilage of ankylosing spondylitis facet joints. Percentage of positively stained chondrocytes among all chondrocytes [%] and representative immunohistochemical staining of BMP-2 (**a**) and BMP-7 (**b**) within the hyaline articular cartilage of facet joints from controls (CO; *A–B upper pictures*), patients with ankylosing spondylitis (AS; *A–B middle pictures*) and osteoarthritis (OA; *A–B lower pictures*); original magnification 40× and 400×; ns = not significant *p* >0.05; ***= *p* <0.001; *= *p* <0.05. *BMP* bone morphogenic protein

We also evaluated the expression of BMP-2 and BMP-7, which on the one hand are growth factors promoting late hypertrophic differentiation of chondrocytes in vitro and on the other hand are important for the maintenance of articular cartilage [26]. The percentage of chondrocytes expressing BMP-2 and BMP-7 was significantly lower in AS joints (mean ± SD: BMP-2 = 1.0 ± 1.3 %, BMP-7 = 2.2 ± 2.5 %) compared to CO and OA joints (OA: BMP-2 = 13.9 ± 12.9 %, BMP-7 = 13.2 ± 8.8 %; CO: BMP-2 = 14.6 ± 8.6 %, BMP-7 = 7.6 ± 5.5 %; Fig. 4a and b) while the percentage of positive chondrocytes was not significantly different between OA and CO joints.

### Reduced Sox9 expression in AS joints

**Fig. 5:**
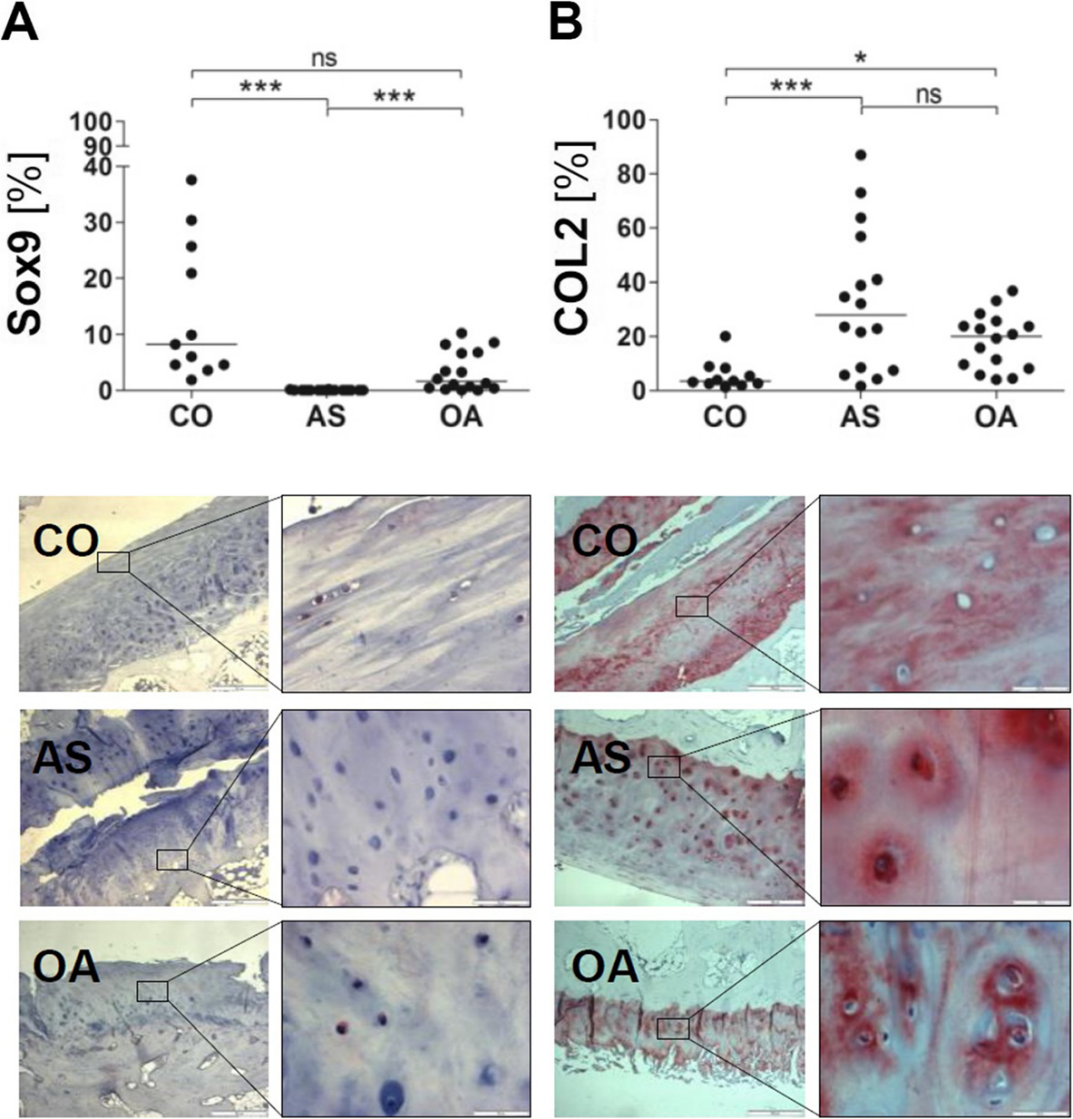
Reduced Sox9 expression but increased COL2 expression in ankylosing spondylitis facet joints. Percentage of positively stained chondrocytes among all chondrocytes [%] and representative immunohistochemical staining of Sox9 (**a**) and COL2 (**b**) within the hyaline articular cartilage of facet joints from controls (CO; *A–B upper pictures*), patients with ankylosing spondylitis (AS; *A–B middle pictures*) and osteoarthritis (OA; *A–B lower pictures*); original magnification 40× and 400×; ns = not significant *p* >0.05; ***= *p* <0.001; *= *p* <0.05. *COL2* type II collagen, *Sox9* sex determining region Y (SRY)-box 9

To further characterize the cartilage phenotype in AS joints we also analyzed the expression of Sox9 as well as COL2 synthesis as additional indicators of chondrocyte integrity. As a result, we observed a drastic reduction in the number of Sox9-positive chondrocytes in AS joints compared to CO joints while Sox9 expression was not altered in OA joints (mean ± SD: AS = 0.02 ± 0.06 %, CO = 13.9 ± 12.5 %; OA 3.3 ± 3.5 %; Fig. 5). In contrast, the number of COL2-expressing chondrocytes was significantly increased in AS joints compared to CO and OA joints (mean ± SD: AS = 32.7 ± 26.1 %, CO = 5.7 ± 5.4 %; OA 18.4 ± 10.3 %; Fig. 5).

## Discussion

In this immunohistochemical analysis we performed a detailed characterization of the phenotype of the cartilage in facet joints of AS patients to decipher the mechanism of joint fusion and joint remodeling observed in these joints. To determine if reactivation of the endochondral pathway of ossification, which leads to sacroiliac joint fusion and ankylosis in the transgenic TNF mouse model after inhibition of the *wnt* pathway inhibitor DKK-1 [6], is involved in the remodeling process, we analyzed in situ the expression of established markers of chondrocyte hypertrophy. However, we neither found an activation of the *wnt* pathway according to beta-catenin expression nor an upregulation of COL10 or MMP13 expression in chondrocytes of AS facet joints. Thus, no conversion of chondrocytes into a hypertrophic phenotype was detected in AS joints, which was, however, readily detectable in the cartilage of OA joints in our analysis and in reports by others [25, 27–31]. Only for Runx2 expression, we observed a tendency toward higher expression specifically at early stages of joint remodeling, which was, however, not accompanied by enhanced expression of COL10 and MMP13. The limited availability of stage I joints, i.e., joints in a prefusion state, is a limitation of the study. However, also in stage II joints, which contain areas with fused cartilage and areas with nonfused cartilage, no evidence of chondrocyte hypertrophy was observed, which strengthens the view that chondrocyte hypertrophy is neither involved in the process of cartilaginous joint fusion nor at later stages of joint remodeling in AS joints.

Surprisingly, the number of beta-catenin-expressing chondrocytes did not inversely correlate with the expression of *wnt* antagonists, neither under physiological (CO) nor under pathological conditions (AS, OA). In fact, the percentage of beta-catenin-expressing chondrocytes was low in AS joints even though wif-1 expression was also strongly reduced in these joints. In OA joints, increased expression of beta-catenin was observed in the absence of dysregulated *wnt* inhibitor expression, suggesting that rather the expression of *wnt* activators than the expression of the *wnt* inhibitors may control chondrocyte hypertrophy under these conditions.

Apart from wif-1, the expression of BMP-2 and BMP-7 was also strongly reduced in AS facet joints. BMPs are not only involved in chondrocyte differentiation but also critical mediators for cartilage homeostasis. Thus, impaired expression of BMPs was shown to promote cartilage degeneration in arthritis models [17] and accompanied dedifferentiation of chondrocytes in vitro [32]. In addition, a reduction in wif-1 expression was also shown to promote cartilage damage in experimental arthritis [33] suggesting that the reduction of wif-1, BMP-2 and BMP-7 expression together with reduced expression of Sox9, a master regulator of chondrocyte differentiation, in the cartilage in AS joints may be considered as signs of cartilage degeneration.

In contrast to the reduction of Sox9, wif-1, BMP-2 and BMP-7 expression, the expression of COL2 was strongly increased in AS joints compared to controls. Induction of COL2 expression is observed in in vitro cultures of human chondrocytes in the presence of reduced oxygen levels suggesting that COL2 induction reflects counterregulation in response to tissue stress [34]. Interestingly, MMP13 expression was reduced in the presence of hypoxia which also led to reversion of the hypertrophic phenotype of OA chondrocytes [34]. Thus, the enhanced COL2 expression in AS cartilage might indicate a response of the cartilage to tissue stress.

In summary, our study found no evidence of chondrocyte hypertrophy in AS facet joints as a putative mechanism which could support cartilage fusion and subsequent joint remodeling. We rather found further signs of cartilage degeneration such as severe reduction in Sox9 expression and a decreased expression of BMP-2 and BMP-7, in addition to the previously reported proteoglycan loss and increase in chondrocyte apoptosis in the cartilage of AS joints [4]. The low expression of beta-catenin in AS facet joints underlines this assumption, because Zhu et al. showed that activation as well as inhibition of beta-catenin signaling in articular chondrocytes results in articular cartilage destruction [8, 35]. Together with several other reports describing extensive cartilage degeneration in AS joints according to histomorphology [36–38], our immunohistological characterization of chondrocytes suggests that cartilage degeneration is a hallmark of the joint pathology in AS joints and may promote cartilage fusion. Factors that promote this degeneration are still enigmatic and have to be studied in the future.

## Conclusions

Our analysis shows that cartilaginous fusion and ankylosis of facet joints in AS patients is not mediated by chondrocyte hypertrophy but rather promoted by cartilage degeneration.

## Abbreviations

AS: ankylosing spondylitis
BMP: bone morphogenic proteins
CO: controls
COL10: type X collagen
COL2: type II collagen
DKK-1: dickkopf-1
MMP: matrix metalloproteinase
NSAIDs: nonsteroidal anti-inflammatory drugs
OA: osteoarthritis
Runx2: runt-related transcription factor 2
SD: standard deviation
Sox9: sex determining region Y (SRY)-box 9
TNF: tumor necrosis factor
wif-1: wingless inhibitor factor-1
*wnt*: wingless
